# Biogeography of functional trait diversity in the Taiwanese reef fish fauna

**DOI:** 10.1002/ece3.4771

**Published:** 2018-12-26

**Authors:** Vianney Denis, Jian‐Wen Chen, Qi Chen, Yunli Eric Hsieh, Yuting Vicky Lin, Ching‐Wei Wang, Hui‐Yu Wang, Nicolas Sturaro

**Affiliations:** ^1^ Institute of Oceanography National Taiwan University Taipei Taiwan; ^2^ Institute of Fisheries Science National Taiwan University Taipei Taiwan; ^3^ Present address: Laboratory of Oceanology FOCUS, University of Liège Liège Belgium

**Keywords:** functional diversity, high‐latitude, ichthyofauna, Kuroshio, marginality, traits

## Abstract

The richness of Taiwanese reef fish species is inversely correlated to latitude as a direct consequence of the abiotic environment and its effects on benthic habitats. However, to date, no studies have investigated the variations in the diversity of traits (*FD*) linked with the role of these fishes in the ecosystem. *FD* is usually considered more sensitive than species richness in detecting early changes in response to disturbances, and therefore could serve as an indicator of ecological resilience to environmental changes. Here, we aim to characterize *FD* in the Taiwanese reef fish fauna and to document its regional variations. Six traits were used to categorize the 1,484 reef fish species occurring in four environmentally contrasted regions around Taiwan. The number of unique trait combinations (*FEs*), their richness (*FRic*), their redundancy (*FR*), their over‐redundancy (*FOR*), and their vulnerability (*FV*) were compared among these regions. Overall, 416 *FEs* were identified. Their number decreased from south to north in step with regional species richness but *FRic* remained similar among regions. *FR* and *FOR* were higher to the south. At the local scale, variations in *FEs* and *FRic* are in concordance with the worldwide pattern of *FD*. High‐latitude, impoverished fish assemblages, offer a range of trait combinations similar to diversified tropical assemblages. Increasing diversity in the latter mainly contributes to raising *FR* and supports already over‐redundant entities. High vulnerability makes many combinations highly sensitive to species loss, and was higher at intermediate latitudes when using a fine resolution in trait categories. It suggests that the loss of *FEs* may first be characterized by an increase in their vulnerability, a pattern that could have been overlooked in previous global scale analyses. Overall, this study provides new insights into reef fish trait biogeography with potential ramifications for ecosystem functioning.

## 1 INTRODUCTION

Rising seawater temperatures from climate change are pushing tropical marine organisms close to their upper thermal limits (Walther et al., 2002) and transforming coral reef ecosystems (Hughes et al., 2018; Stuart‐Smith, Brown, Ceccarelli, & Edgar, 2018). At a global scale, this is commonly associated with a flattening of coral reefs (Alvarez‐Filip, Dulvy, Gill, Côté, & Watkinson, 2009; Bozec, Alvarez‐Filip, & Mumby, 2015; Wilson et al., 2010), which further reduces the availability of niche spaces upon which a large number of living organisms depend (Graham & Nash, 2013). These modifications notably alter the structure of reef fish assemblages as well as their trophic interactions (Darling et al., 2017; Graham et al., 2007; Pratchett, Hoey, Wilson, Messmer, & Graham, 2011; Richardson, Graham, Pratchett, Eurich, & Hoey, 2018; Wilson et al., 2010). The susceptibility of fish species to extirpation is usually nonrandom (Graham et al., 2011) and benefits generalists, which today seem able to proliferate on reefs worldwide (Munday, 2004; Richardson et al., 2018). This occurs to the detriment of a small proportion of species with often prominent roles in maintaining ecological processes (Mouillot, Bellwood, et al., 2013a), although generalists can play influential roles too. The loss of functionally important fishes can eventually decrease the capacity of the ecosystems to recover from, or resist transitions to, alternative states, which may have only a limited capacity to sustain original ecosystem services (Chong‐Seng, Nash, Bellwood, & Graham, 2014; Fung, Seymour, & Johnson, 2011; Hoey & Bellwood, 2011; Hughes et al., 2017; Pratchett, Hoey, & Wilson, 2014). Consequently, a better understanding of the diversity in functional groups, as well as the functional relationships between coral habitats and associated fish assemblages are of critical importance in managing reefs and prioritizing conservation efforts (Richardson et al., 2018).

Reef fishes represent a dominant structuring force on coral reefs (Raymundo, Halford, Maypa, & Kerr, 2009), controlling the distribution and abundance of many taxa such as mollusks and algae, as well as hard corals (Ceccarelli, Jones, & McCook, 2011; Jompa & McCook, 2002; McClanahan, 1994). Indeed, several critical functional groups of fishes have now been identified that enhance the prospects of recovery toward initial conditions after disturbances (*e.g.*, Bellwood, Hughes, & Hoey, 2006a; Cheal et al., 2010; Graham, Jennings, MacNeil, Mouillot, & Wilson, 2015). For example, herbivorous fishes can mediate the growth, and sediment trapping abilities of algal turf (Goatley, Bonaldo, Fox, & Bellwood, 2016; Tebbett, Goatley, & Bellwood, 2017), which is likely to benefit coral recruitment (Birrell, McCook, & Willis, 2005). In some other cases, predatory fishes may structure reef communities through top‐down control, potentially preventing trophic cascades (Dulvy, Freckleton, & Polunin, 2004; but see Casey et al., 2017 and discussion in Strong, 1992).

Recently, the unique combination of traits (characteristics) describing the role of fish species in the ecosystem has tentatively been proposed as synthetic representation of the functions they performed (Mouillot et al., 2014). This representation is commonly considered a pragmatic description of their potential niche and corresponds to any feature at the individual level that can affect and determine how species use resources and how they interact with each other (Cadotte, Carscadden, & Mirotchnick, 2011). To date, traits responding to environmental variations and focusing on individual performance (“functioning traits”; see Denis, Ribas‐deulofeu, Sturaro, Kuo, & Chen, 2017) have been largely overlooked in this definition. Instead, six categorical traits related to food acquisition and locomotion, considered pertinent to representing key facets of fish ecology (size, diet, mobility, gregariousness, period of activity, and vertical position in the water column) have been widely employed (Mouillot, Bellwood, et al., 2013a; Mouillot et al., 2014). The depiction of the diversity of those traits through discrete values has gained in popularity, and sometimes adjusted, for providing a better insight into ecosystem functions beyond that given by species diversity measures (Richardson et al., 2018). Although, several important caveats undermine this relationship (Brandl, Emslie, & Ceccarelli, 2016) and this approach is perfectible (Villéger, Brosse, Mouchet, Mouillot, & Vanni, 2017). However, functional trait‐based approach and, more precisely, the characteristics of the unique trait combinations, represents today a benchmark for assessing the diversity of functional traits in the communities (Mouillot, Graham, et al., 2013b).

Diversity of traits (*FD*), the range of things that organisms do in communities and ecosystems (Petchey & Gaston, 2006), is directly connected to ecosystem processes and stability (Chapin et al., 2000; McCann, 2000; Purvis & Hector, 2000). *FD* is measured in several ways (Cadotte et al., 2011), and its monotonic response to disturbance usually makes it a better indicator than the traditional taxonomic measurements of early changes affecting reef communities (D'Agata et al., 2014; Mouillot, Graham, et al., 2013b). As an example, focusing on trophic trait, Bellwood, Hughes, Folke, and Nyström (2004) examined fish species richness in 14 pre‐determined categories comparing Caribbean and Australian reefs. The Caribbean assemblage was characterized by a lower redundancy (*i.e.,* average number of species among categories) than the Australian, which depicted the higher vulnerability of Caribbean reef ecosystems for the trait of interest.

By including morphological information into a trait morphospace, Bellwood, Wainwright, Fulton, and Hoey (2006b) further confirmed high versatility in trophic categories characterizing coral reef fish diversity. In most feeding guilds, this was supported by a considerable overlap in the occupation of the morphospace by different morphologies. Further extension to behavioral traits reinforced that a small number of key combination of traits could provide the basic ecological structure of reef fish assemblages (Guillemot, Kulbicki, Chabanet, & Vigliola, 2011). This is illustrated on a global scale by a high redundancy that is disproportionately packed into few trait combinations (over‐redundancy). In a nonrandom assembly, it leaves a large number of entities supported by only one species, thus increasing the overall vulnerability of trait combinations in the reef fish fauna and the potential sensitivity of *FD* to the loss of a few species (Mouillot et al., 2014). Surprisingly, *FD* is revealed to be relatively stable across biogeographical provinces despite their high variability in species richness (Mouillot et al., 2014). Hence, impoverished fish faunas such as those from the Tropical Eastern Pacific and the Atlantic could maintain the range of ecological processes necessary for the growth and persistence of tropical reefs since they share most of the key functions of reefs having richer fauna (Bellwood et al., 2004; Johnson, Jackson, & Budd, 2008). Higher diversity in other provinces mainly contributes to over‐redundancy, leaving *FD* highly vulnerable to species loss.

Biogeographical provinces primarily correspond to geographical areas with distinctive fish faunas (Briggs & Bowen, 2012; Kulbicki et al., 2013). Actually, their definitions confound environmental parameter operating at a more local scale that control species distribution. Abiotic parameters such as temperature, light, and hydrodynamic regime are important variables defining ecoregions (Spalding et al., 2007) and strongly determine the regional composition of coral communities (Kleypas, McManus, & Meñez, 1999). Fish diversity (across reef habitats) varies on a latitudinal gradient together with changes happening in benthic assemblages (Bellwood & Hughes, 2001; Hillebrand, 2004; Mora, Chittaro, Sale, Kritzer, & Ludsin, 2003; Tittensor et al., 2010). Those changes can modify the trophic structure of an ecosystem (Ferreira, Floeter, Gasparini, Ferreira, & Joyeux, 2004) and eventually influence regional *FD*. However, there is always the possibility that a local variation has been largely overlooked in previous studies.

Taiwan is located to the north of the East Indies Triangle, the region hosting the highest marine diversity in the world (Briggs, 2005). It encompasses tropical and subtropical latitudes and spans the transition of two marine realms where three provinces and ecoregions converge (Spalding et al., 2007). The Taiwanese reef fish fauna is highly diverse (Allen, 2008), and species richness decreases steeply from southeast to northwest in correlation to the patterns in sea surface temperatures that shape reef habitats (Dai, Soong, Chen, Fan, & Hsieh, 2005). Therefore, Taiwan constitutes an ideal location for investigating the potential effects of environmental settings on the *FD* of reef fish assemblages. The objectives of this study were to (a) compare *FD* among the main regions of coral development around Taiwan with respect to prevailing environmental conditions, and (b) examine the local spatial patterns of *FD* of Taiwanese reef fish fauna in comparison to global patterns. To achieve this, the present study compares the richness, redundancy, over‐redundancy, and vulnerability of the unique functional trait combinations identified in four coral regions around Taiwan characterized by contrasting environmental conditions.

## 2 MATERIALS AND METHODS

### 2.1 Reef fish fauna

**Figure 1:**
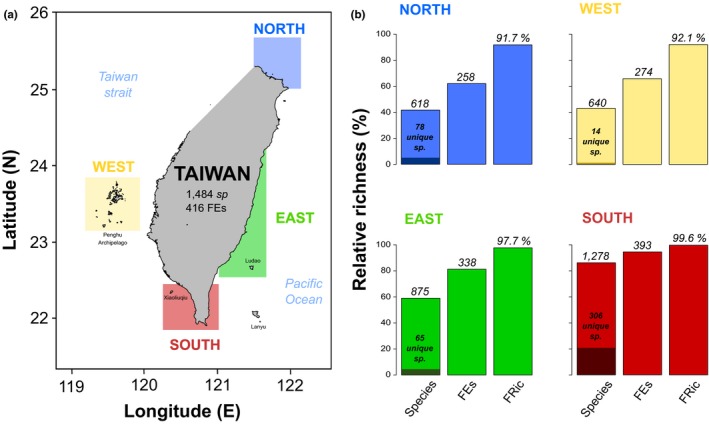
(a) The four studied regions around Taiwan. (b) Their respective species richness and number of unique species, unique trait combinations (*FEs*), range of unique trait combinations (*FRic*). The y‐axis is the percentage of richness in all bar plots

Our study focuses on four well‐supported reef fish assemblages (so‐called regions, Figure 1a): North, East (including Ludao), West (Penghu Archipelago), and South of Taiwan (including Xiaoliuqiu). This distinction is justified by environmental conditions producing contrasting benthic assemblages (Chen, 1999; Chen & Shashank, 2009; Ribas‐Deulofeu et al., 2016) and hosting distinct reef fish assemblages (Shao, Chen, & Wang, 1999). The warm waters of the Kuroshio Current flow from the southern point of Taiwan along its east coast toward the Ryukyu Archipelago, pushing tropical organisms (*e.g.,* scleractinian corals; Chen, 1999) northward. Accordingly, fringing reefs only occur on the East and South coasts of Taiwan, where average monthly seawater temperatures remain above 20°C. In contrast, the frequent occurrence of waters lower than 18°C in winter prevents the accretion of reefs to the West and the North of Taiwan (Chen, 1999; Kleypas et al., 1999; Wang & Chern, 1988). There, only non‐reefal and less diversified coral assemblages develop directly on the basalt substrate that shapes the coastline. From a benthic survey throughout 2011–2012 at selected sites, coral cover at 5 m in depth were estimated to 24.1% at Kenting (South), 39.6% at Green Island (East), 21% at Penghu (West), and 18.8% to the North of Taiwan (Ribas‐Deulofeu et al., 2016).

Reef fish composition in these four regions were obtained by corroborating information from Taiwan FishBase (Shao, 2018), FishBase (Froese & Pauly, 2018), and additional references documenting local reef fish diversity (Chen, 2004a, 2004b; Chen, Jan, Kuo, Huang, & Chen, 2009; Chen, Shao, Jan, Kuo, & Chen, 2010; Shao et al., 2008). Here, a fish was considered as a coral reef species if it is partly or strictly associated, during its lifetime, to shallow coastal coral habitat, including the non‐reefal environments to the West and the North of Taiwan. For each species, its synonymy and status validity were checked in the World Register of Marine Species (WoRMS Editorial Board, 2018), and only valid species were used for further analysis. This step proved to be of critical importance in the preparation of data extracted from databases.

### 2.2 Trait selection

Our final list of reef fish species among the four regions in Taiwan encompassed 1,484 species. A species was considered as unique to a region when it was absent from the others. Each species was classified into six categorical traits reflecting its possible functions (the role of the species) in the ecosystem: size, diet, mobility, gregariousness, period of activity, and vertical position in the water column (following Mouillot et al., 2014). These traits and their levels are detailed in Supporting Information Appendix S1. Their relevance in describing reef fish functions has been previously evaluated tentatively by Mouillot et al. (2014). The strength of the application of these six traits depends on the extent to which these traits really are indicative of functional attributes (Mouillot, Graham, et al., 2013b). However, their extensive use in the broader field of reef fish functional ecology does not imply their links to ecosystem functioning are clear (see Beauchard, Veríssimo, Queirós, & Herman, 2017; Villéger et al., 2017 for discussion). Species trait information was extracted from databases and/or relevant references (Froese & Pauly, 2018; Shao, 2018). Because trait values can differ among sources, priority was first given to FishBase, followed by Taiwan FishBase, then other references. Missing species trait values were infilled with specific literature on the given species or with the expertise of the authors considering the phylogenetic position of the species. The final dataset provides a species list, regional distributions, and selected traits.

### 2.3 Trait entities, space, and indices

#### 2.3.1 Trait entities

Functional trait entity (*FE*) was defined as a unique combination of categorical trait values. Theoretically, the six traits and their categories yield a total number of 5,670 unique combinations. Yet many combinations do not occur and only 646 FEs (11.4%) were defined based on an analysis of global reef fish fauna (Mouillot et al., 2014).

#### 2.3.2 Trait space

A dissimilarity (trait) matrix was produced by a pairwise comparison of the *FEs* using Gower distance (S15). S15 has the advantage of being suitable for mixed (continuous and categorical) variables, and is therefore applicable here. A Principal Coordinates Analysis (PCoA) was then computed on the basis of this trait matrix applying a Caillez correction to correct any potential negative eigenvalues generated (Cailliez, 1983). Euclidean distances among *FEs* on the first four axes of this PCoA were firmly correlated with the initial Gower matrix (Mantel test, *r* = 0.77, *p* < 0.001), and the addition of an extra axis only marginally increased this resolution. Therefore, information on the first four axes was considered the most pragmatic representation of variation among *FEs*. The coordinates of *FEs* on these axes were used to represent synthetic trait space (to visualize relationship among *FEs*) and later calculate *FD* indices.

#### 2.3.3 Trait diversity indices

In addition to the number of *FE*s, the range of unique trait combinations (*FRic,* Mason, Mouillot, Lee, & Wilson, 2005) was computed for each region. *FRic* was calculated in the four dimensions of our trait space. It expresses for the fish assemblage in each region the proportion of the volume of the convex hull (%) that the species occupied. In addition, we described the four reef fish assemblages around Taiwan based on the characteristics of their *FEs*. The redundancy of trait combinations (*FR*) corresponds to the average number of species sharing similar sets of traits (Equation 1); the vulnerability of trait combinations (*FV*, %) represents the proportion of trait combinations supported by only one species (Equation 2); and the over‐redundancy of trait combinations (*FOR*, %) indicates the percentage of species in *FEs* having more species than expected from *FR* (Equation 3). Following Mouillot et al. (2014), those indices are expressed as:

(1) $$FR=\frac{\sum_{i=1}^{\mathrm{FE}}n_{i}}{\mathrm{FE}}=\frac{S}{\mathrm{FE}}$$

(2) $$FV=\frac{FE-\sum_{i=1}^{\mathrm{FE}}\text{min}\left(n_{i}-1,1\right)}{\mathrm{FE}}$$

(3) $$FOR=\frac{\sum_{i=1}^{\mathrm{FE}}\left[\max\left(n_{i},FR\right)-FR\right]}{S}$$

where *FE* is the total number of trait entities, *S* is the total number of fish species, and is the number of species in a trait entity .

*FV* and *FOR* are influenced by the number of species, the number of *FEs*, and the distribution of species among *FEs*. Therefore, to test whether the observed values were significantly different from theoretical values, the observed values of *FV* and *FOR* were compared with the null hypothesis that species were distributed randomly in each *FE*. Briefly, for each of the four study assemblages, we randomly assigned species in the previously determined number of *FEs *(ensuring that at least one species occupied each *FE*). In this way, we simulated 9,999 assemblages and computed *FV* and *FOR* in each of them. We compared observed *FV* and *FOR* values to theoretical values with a bilateral test (*α* = 0.05) and further recognized how significantly different they were from a random distribution by examining the standardized effect of size. For instance, if the observed values of *FV *and *FOR *were higher than simulated, it indicated that the distribution of species was concentrated within particular *FEs*, leaving a number of *FEs* supported by only a single species.

The sensitivity of our results to the extent of trait categories was further assessed using a crude categorization following Mouillot et al. (2014). It consisted of reducing trait categories as detailed in Supporting Information Appendix S1, rerunning all the analyses on this basis, and comparing the results with the ones from the finer resolution in traits categories. Theoretically, the six traits and their crude categorization yield a total number of 216 unique combinations.

All data analyses were performed in R software (v3.4.3, R Core Team, 2017) using worms (Holstein, 2018), ape (Paradis, Claude, & Strimmer, 2004), cluster (Maechler, Rousseeuw, Struyf, Hubert, & Hornik, 2017), and FD (Laliberté & Legendre, 2010; Laliberté, Legendre, & Shipley, 2014) packages as well as the functions “quality_funct_space” and “multidimFD” (Mouillot, Graham, et al., 2013b) available at http://villeger.sebastien.free.fr/Rscripts.html (last accessed 2018/07/19).

## 3 RESULTS

### 3.1 Fish richness and unique trait combinations

Fish species richness ranges between 618 species in the North to 1,278 species in the South, which respectively represent 41.6% and 86.1% of Taiwan's total richness (Figure 1b). The South has the highest number of unique species (306 species, 23.9% of the regional richness), followed by the North and East with 78 (12.6%) and 65 (7.4%) unique species, respectively. The western region hosts only 14 unique species (2.2% of the regional richness). The classification of those species from all four regions into six traits yields a total of 416 *FEs* representing 7.3% of the total theoretical number of unique combinations (*i.e*., 5,670 combinations). The number of *FEs* follows the species richness pattern and is higher in the South (393 *FEs*) compared to the East (338 *FEs*), West (274 *FEs*), and North (258 *FEs*; Figure 1b).

### 3.2 Trait space and richness

Most of the variation in our Gower matrix comparing the *FEs *was caught in the first four axes of our trait space (Mantel test, *R* ^2^ = 0.58, *p* < 0.001). It also reduced the mean squared deviation considerably between the initial distance and, the standardized final distance in the trait space (quality_funct_space function, mSD = 0.01). Therefore, the dispersions among *FEs *and the computation of *FRic* were considered in four dimensions. The positions of our 416 *FEs* were determined by unique combinations of trait values (Supporting Information Appendix S2). *FRic* represents the hypervolume delimited by the most extreme entities in these four dimensions and was computed for each region as the proportion that local fauna occupied (Supporting Information Appendix S3). Regional *FRic *are 99.6% in the South, 97.7% in the East, 92.1% in the West, and 91.7% in the North (Figure 1b). Therefore, despite variable species richness and *FEs*, the range of unique trait combinations supported by each assemblage, remain relatively stable among regions with only a few differences noticeable in the 3rd and 4th dimensions of our trait space (Supporting Information Appendix S3). Similar parts of the trait space were lost in the West and the North explaining the slightly lower *FRic* values in these regions.

### 3.3 Vulnerability, redundancy, and over‐redundancy of trait combinations

**Figure 2:**
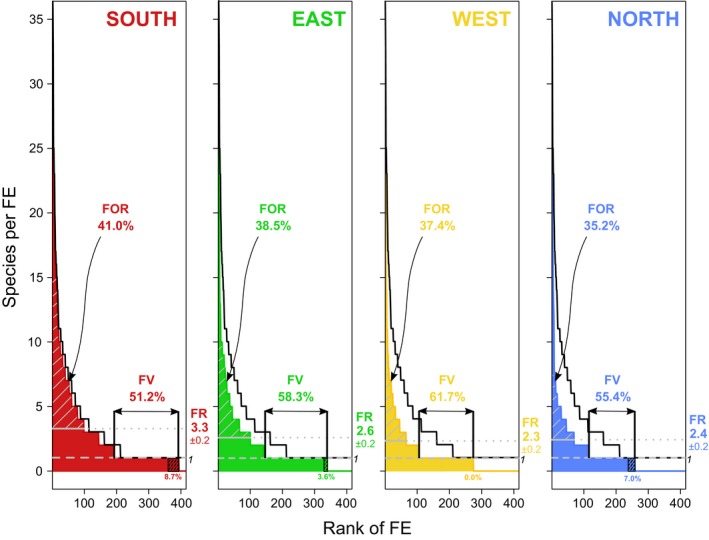
Distribution of species among unique trait combinations (*FEs*) with their redundancy (*FR*), vulnerability (*FV*), and over‐redundancy (*FOR*) among regions around Taiwan. *FR* is the mean number of species among all *FEs*. *FV* is the percentage of *FEs* possessing only one species. *FOR* is the percentage of species contributing to the over‐representation of some sets of traits (*i.e*., gray oblique lines). The black stair line corresponds to the distribution of species in *FEs* in the overall Taiwanese reef fish fauna. The black oblique dashed line area represents the contribution of unique species to *FV*

The distribution of fish species by *FEs *displays a characteristic positive skewness in all four regions (Figure 2). Testing the species richness‐related indices (*i.e*., *FOR* and *FV*) against null models (Supporting Information Appendix S4) reveals that the distributions observed are nonrandom (*p* < 0.001) and that species are disproportionally packed into a low number of *FEs*. The average (±*SE*) number of species per *FE *(*FR*) is the highest in the South (3.3 ± 0.2 species) and the lowest in the West (2.3 ± 0.2 species; Figure 2). *FOR* indicates that about 42.4% of the species contribute to the over‐representation of some *FEs* at the national scale. *FOR* ranges between 41.0% in the South and 35.2% in the North, with intermediate levels of 38.5% and 37.4% for the East and West, respectively (Figure 2). Notably, many *FEs *are left without insurance, as almost half (49.0%) are supported by only a single species at the national scale. At a regional level, *FV* values are lower in the South (51.2%) and intermediate in the North (55.4%). *FV* reaches maximum values at intermediate latitudes with 61.7% and 58.3%, in the West and East of Taiwan, respectively (Figure 2). Unique species contribute 8.7% in the South, 3.6% in the East, 0.0% in the West, and 7.0% in the North, to *FV* (Figure 2).

### 3.4 Sensitivity analysis

The reduction of trait categories decreased the number of *FEs* by 4.4‐fold (22.6%) in the overall Taiwanese reef fish fauna (94 vs. 416 *FEs*). As a consequence, it increased the redundancy in trait combinations (15.8 ± 2.7), but species remained disproportionally packed into a low number of *FEs* (*FOR*: 49.4%) leaving many of them (18.1%) with no insurance. Crude categorization slightly increases *FOR* (+7.0%) and decreases *FV* (−30.9%). Regionally, it reduced the number of *FEs* by 4.2‐fold in the South, 3.7‐fold in the East, and 3.1‐fold in both the West and the North of Taiwan (Table 1). Patterns in regional variation of *FE* and *FRic*, *FR* and *FOR* are consistent with the one using a finer resolution in trait categories. However, *FV* was found to increase with latitudes whereas it was maximum at intermediate latitudes using a finer resolution in trait categories. None of the unique species at a regional level contributes to the *FV* defined using the coarser categorization (Supporting Information Appendix S4).

**Table 1:** Sensitivity analyses

	*FEs *(% richness)	*FRic *(%)	*FR*	*FOR *(%)	*FV *(%)
North	83 (88.3)	91.0	7.5	46.3	30.1
West	87 (92.6)	93.9	7.4	48.6	28.7
East	91 (96.8)	98.5	9.6	47.0	22.0
South	94 (100)	100	13.6	49.1	20.2

Note

Unique trait combinations (*FEs*), range of unique trait combination (*FR*) as well as their redundancy (*FR*), vulnerability (*FV*), and over‐redundancy (*FOR*) among regions around Taiwan after reducing the number of trait categories. Overall, this coarse categorization reduced the number of possible *FEs* by 4.4 fold (94 vs. 416 using a fine resolution in trait categories).

## 4 DISCUSSION

Around Taiwan, the reef fish fauna encompasses 1,484 species, which is exceptionally high considering the small overall area of reefs (~940 km^2^) around the island (Allen, 2008). It yields 416 *FEs,* which correspond to 7.3% of the theoretical maximum possible. In comparison, using the same traits and categories, the world reef fish fauna (6,316 species) yields 646 *FEs*, and 11.4% of the theoretical combinations (Mouillot et al., 2014). Taiwan is located at the northern limit of the Central Indo‐Pacific province, in which the fish fauna yields 468 *FEs *across 3,600 species (Mouillot et al., 2014). Although there is a two‐fold difference in species richness between the overall province and Taiwan, there is an 89% similarity in the total number of *FEs* observed. This similarity in FEs suggests that the poorer Taiwanese reef fish fauna may be able to maintain the ecological processes necessary for the growth and persistence of reef ecosystems (Bellwood et al., 2004; Johnson et al., 2008).

The four studied regions also had similar *FRic *values despite exhibiting large differences in species richness. The four regions fill between 91.7% and 99.6% of the trait space defined at the national scale. The lowest values to the West and the North are mostly explained by the loss of some given *FEs*, for which the possible link with prevailing environmental conditions warrants further investigation. Congruent with patterns observed among global biogeographic provinces (Mouillot et al., 2014), variation in species richness or in the number of *FEs,* seems to have little influence on *FRic* levels estimated in the four fish assemblages assessed. This pattern is especially interesting in light of the scale of our study and the contrasting coral richness among the four regions (Dai et al., 2005).

While coral trait diversity is also remarkably conserved among global biogeographic provinces and along gradients of species richness (McWilliam et al., 2018), *FRic *that characterizes coral assemblages around Singapore responds strongly to patterns in species distribution (Wong et al., 2018). Based on the functional relationships identified between coral and reef fish assemblages among sites (Darling et al., 2017), we could have expected a similar reduction of *FRic* in marginal locations where coral richness is impoverished. We hypothesized that regional consideration of species occurrence (gamma trait diversity–regional pool) could confound fine variation in *FRic* (alpha trait diversity–local pool), which might be relevant in interpreting local, and more specifically, human‐mediated loss of *FD* (D'Agata et al., 2014). Site‐specific, local surveys of reef fish abundance or biomass (Stuart‐Smith et al., 2013) may provide further insight into the *FRic* pattern within regions, in particular its responses to local degradation in benthic assemblages, and their associated traits, as observed around Singapore (Wong et al., 2018).

Environmental conditions in the North and the West of Taiwan are marginal for corals (Dai & Horng, 2009) and thus limiting for reef accretion (Kleypas et al., 1999). Although these two regions only host about half of the fish species recorded from the South of Taiwan, they sustain a relatively high proportion (66% and 70% for the North and West, respectively) of the total number of *FEs* observed in the South. It suggests that these communities could have the ability to maintain a wide range of ecological processes present in more tropical locations. Consequently, these two impoverished regions have the lowest *FR* and *FOR* values. Even where these values were highest in the South (averaging 3.3 ± 0.2 species per *FE* and 41.0%, respectively), they remain largely below those documented in the Central Indo‐Pacific (7.9 species per *FE* and 57.9%, respectively) and are comparable to values from the impoverished reef fish fauna in the Tropical Eastern Pacific (2.8 species per *FE* and 40.0%, respectively; Mouillot et al., 2014). This supports the notion that the addition of new species does not contribute substantially toward generating new *FEs* (Halpern & Floeter, 2008). Instead, it tends to make existing functional fish assemblages more robust by offering high “insurance” in a small number of *FEs*.

The over‐representation of species in a few *FEs* was further robust to a drastic reduction of the possible number of *FEs* as in the global reef fish fauna (Mouillot et al., 2014). In addition, we demonstrated that the over‐representation of a low number of *FEs* is better preserved than the *FR* along a decreasing gradient of species richness. Despite a similar range of *FEs* offered, it makes the functionality of the fish assemblages from northern, western, and eastern regions potentially more vulnerable and less resilient to disturbances than the southern region.

The location of Taiwan at the periphery of the Central Indo‐Pacific province is reflected in the relatively high vulnerability of its fish fauna. Regional *FVs* ranged between 51.2% and 61.7%, which is actually in the range reported from the Tropical Eastern Pacific province (54.2%) and much higher than the *FV* of the Central Indo‐Pacific (38.5%; Mouillot et al., 2014). This suggests that, independently of the location around Taiwan, at least half of the *FEs* are supported by only one species, leaving many *FEs* without insurance when facing species loss (Mouillot, Bellwood, et al., 2013a). Rare species, both in terms of local abundance and regional occupancy, often support unique and distinct functions (Mouillot, Bellwood, et al., 2013a) that could be critical for the resilience of coral reefs (Bellwood, Wainwright, et al., 2006b). Unfortunately such species are usually the most susceptible to extraction and extirpation (Graham et al., 2011), which may result in the functional homogenization of fish assemblages and a proliferation of generalist species (Munday, 2004; Richardson et al., 2018).

Characteristic of a nonrandom fish assemblage, species loss should result in an increase in *FV* and/or an increase in *FOR* (Halpern & Floeter, 2008). Yet, regional patterns observed at large spatial scales support a global mismatch between species richness and *FV *(Parravicini et al., 2014). Our study further uncovers an interesting pattern in *FV* that macro‐ecological research may have overlooked. Fish assemblages from regions at intermediate latitudes have the highest *FV* values (in the West and East in this case). From the previous observation that *FR* will be more affected by species loss than *FOR,* it suggests that the *FV* of fish assemblages might first increase as species are lost and then drop further after singular functions are removed, ultimately resulting in a functional homogenization of the assemblage (Clavel, Julliard, & Devictor, 2011). In this scenario, functionally unique species could tend to be rarer in tropical than in temperate reef fish assemblages as reported globally by Stuart‐Smith et al. (2013).

A high *FV* has further been interpreted as a pristine state and a baseline for assemblages untouched by human for priority conservation (Quimbayo, Mendes, Kulbicki, Floeter, & Zapata, 2017). In this scenario, *FV* could act as an indicator of a functional “tipping‐point,” a stage prior to more severe functional changes occurring along environmental gradients or from human disturbances. An exciting outcome of these observations will be to test whether *FV* can be applied to temporal community dynamics and if it could serve as a dimension for assessing ecological shifts. However, *FV* level is sensitive to the traits considered as well as their categorization (Mouillot et al., 2014). Overall, *FV* decreases with a crude categorization of traits and raises with latitude, which makes the *FEs* recorded in marginal species‐poor regions more vulnerable to species loss than in richer assemblages. This higher *FV* observed to the North was not caused by unique species. Instead, coarse trait resolution could decrease the susceptibility of identifying *FEs* that are vulnerable and overlook subtle functional changes in the assemblages. Therefore, the resolution in trait categorization and its outcomes should be considered and interpreted carefully according to the scale of changes expected.

In this study, the Taiwanese reef fish fauna offered new insights into the biogeography of reef fish trait diversity, which has recently raised as an important facet of the diversity. It constitutes the first step toward a better understanding of the fishes’ role in the Taiwanese reefal areas. Taiwanese reef fish fauna retains a high proportion of trait combinations defined at the scale of the Central Indo‐Pacific province, but remains highly vulnerable to species loss. Possible overfishing and the consequences of human activities on benthic habitats are likely to have already modified historic diversity (Liu et al., 2009; Ribas‐Deulofeu et al., 2016). Therefore, this study proposes a baseline upon which the gain and loss of unique trait combinations could be immediately assessed after updating information on species richness. The integration of abundance and/or biomass to the current framework (see Chen et al., 2015) would improve the present trait assessment and the functional interpretation of the Taiwanese reef fish fauna. This would further enhance the prospects for exploring functional relationships between reef fish assemblages and coral habitats.

## CONFLICT OF INTEREST

The authors have no conflicts of interest to declare.

## AUTHOR CONTRIBUTIONS

VD designed the experiment. VD and JWC collected the data. VD, JWC, and CWW analyzed the data. VD, JWC, QC, YEH, YVL, CWW, HYW, and NS wrote the paper and revised the drafts.

## DATA ACCESSIBILITY

All data and script for data analysis are available from the Dryad Digital Repository: https://doi.org/10.5061/dryad.838v1j5.
