# Supplementary material for: Biogeography of functional trait diversity in the Taiwanese reef fish fauna
Source: Ecol Evol. 2018 Dec 26;9(1):522–32. doi: 10.1002/ece3.4771 (PMC6342120; doi:10.1002/ece3.4771)
Supplement: Supplementary file 1 [file ECE3-9-522-s001.docx]

**SUPPORTING INFORMATION**

| **trait** | **fine categories** | **crude categories** |
| --- | --- | --- |
| **size (cm)** | **6 categories:**  • 0-7  • 7.1-15  • 15.1-30  • 30.1-50  • 50.1-80  • >80 | **3 categories:**  • 0-15  • 15.1-50  • >50 |
| **diet** | **7 categories:**  • herbivorous-detritivorous  • omnivorous  • macroalgal herbivorous  • invertivorous (sessile invertebrates)  • invertivorous (mobile invertebrates)  • planktivorous  • piscivorous | **3 categories:**  • primary consumers (detritivorous + herbivorous + omnivorous)  • invertivores (sessile and mobile invertivororous, and planktivorous);  • piscivorous |
| **mobility** | 1. **categories:**   • sedentary  • mobile within a reef  • mobile among reefs | **2 categories:**  • sedentary  • mobile |
| **gregariousness** | **5 categories:**  • solitary  • pairing  • small group (3-20 individuals)  • medium group (20-50 individuals)  • large group (>50 individuals) | **3 categories:**  • solitary  • small group (2-20 individuals)  • gregarious (>20 individuals) |
| **period of activity**  **(foraging)** | **3 categories:**  • diurnal  • diurnal-noctural  • nocturnal | **2 categories:**  • diurnal/diurnal-nocturnal  • strictly nocturnal |
| **position water column** | **3 categories:**  • benthic  • bentho-pelagic  • pelagic | **2 categories:**  • benthic/bentho-pelagic  • strictly pelagic |

**Appendix S1. Traits and their levels using the fine and crude categorizations**

**
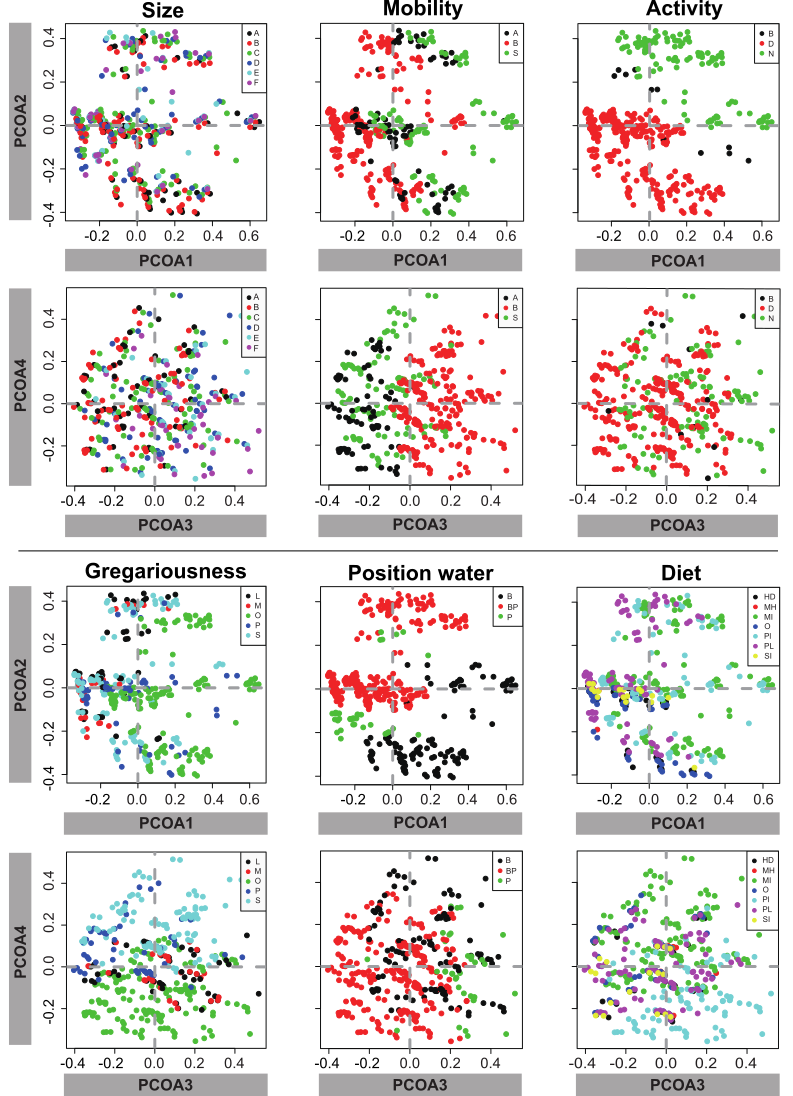
**

**Appendix S2. Distribution and characteristics of the 416 unique trait combinations (*FEs*) in the four first axes of the trait space (PCoA).** Legends for the 6 traits are defined by: **size** *(A: 0-7 cm, B: 7.1-15 cm, C: 15.1-30 cm, D: 30.1-50 cm, E: 50.1-80 cm,* and *F: >80 cm)*; **diet** (*HD: herbivorous-detritivorous, MH: macroalgae herbivorous, SI: invertivorous feeding on sessile invertebrates, MI: invertivorous targeting mobile invertebrates, PL: planktivorous, PI: piscivorous,* and *O: omnivorous*); **mobility** (*S: sedentary, A: mobile within a reef, and B: mobile among reefs*); **gregariousness** (O*: solitary, P: pairing,* and *living in S: small, M: medium, or L: large groups*); **period of activity** (*D: during the day, N: by night,* or *B: both*); **vertical position in the water column** (*B: benthic, BP: bentho-pelagic,* and *P: pelagic*).

**
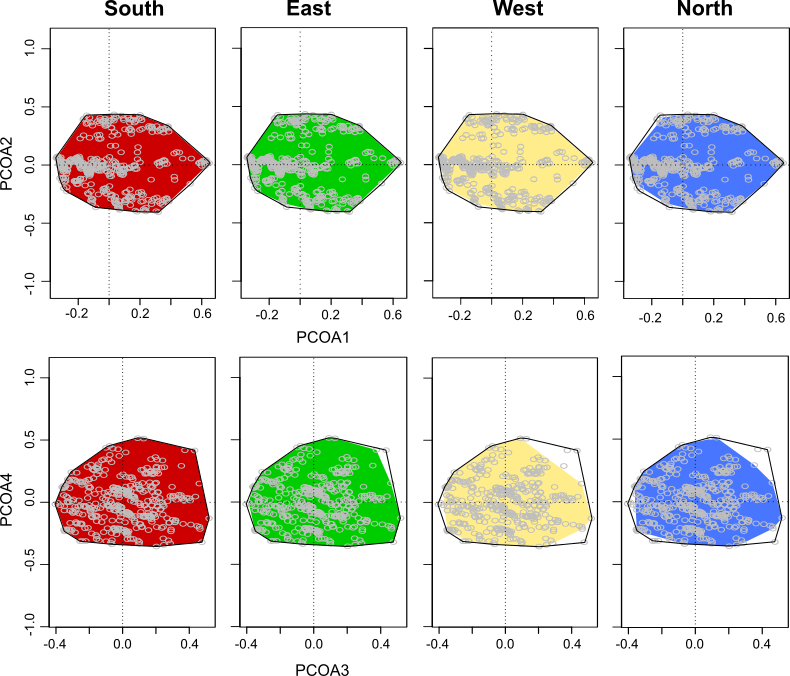
**

**Appendix S3. Trait space filled by regional reef fish species in comparison with the range of trait combinations defined for the overall Taiwanese reef fish fauna.** Richness is represented in the four first axes of the trait space (PCoA). Color areas delineate *FRic* of regional pools, black line depicts *FRic* for the overall Taiwanese reef fish species.

**
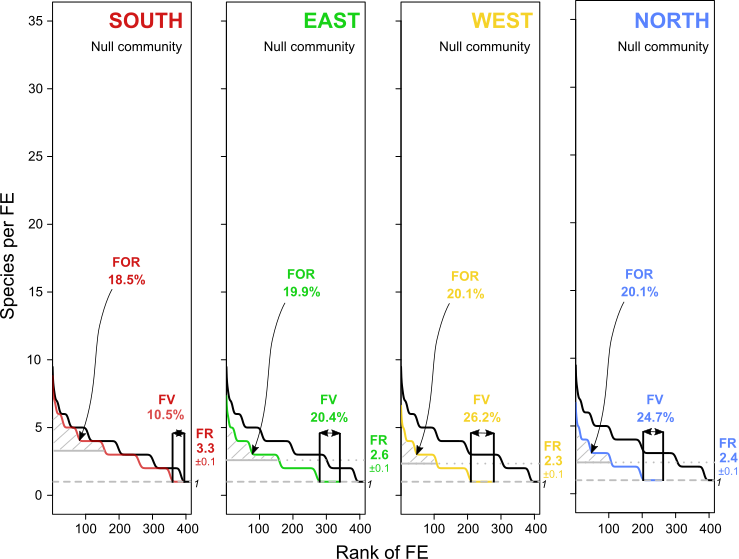
**

**Appendix S4. Theoretical distribution of species unique trait combinations (*FEs*, null community) with indication of their redundancy (*FR*), their vulnerability (*FV*), and their over-redundancy (*FOR*) among regions around Taiwan.** *FR* is the mean number of species among all *FEs*. *FV* is the percentage of *FEs* possessing only one species. *FOR* is the percentage of species contributing to the overrepresentation of some sets of traits *(i.e*. gray oblique lines). The black stair line corresponds to the theoretical distribution of species in *FEs* in the overall Taiwanese reef fish fauna. For the four regions, the contribution of unique species to *FV* is zero.
